# Welfare of Free-Roaming Horses: 70 Years of Experience with Konik Polski Breeding in Poland

**DOI:** 10.3390/ani10061094

**Published:** 2020-06-24

**Authors:** Aleksandra Górecka-Bruzda, Zbigniew Jaworski, Joanna Jaworska, Marta Siemieniuch

**Affiliations:** 1 Department of Animal Behaviour, Institute of Genetics and Animal Breeding, Polish Academy of Sciences, 05-552 Jastrzębiec, Poland; 2 Department of Horse Breeding and Riding, Faculty of Animal Bioengineering, University of Warmia and Mazury, 10-719 Olsztyn, Poland; zbigniew.jaworski@uwm.edu.pl; 3 Department of Gamete and Embryo Biology, Institute of Animal Reproduction and Food Research, Polish Academy of Sciences, 10-243 Olsztyn, Poland; joanna.jaworska11@gmail.com; 4 Department of Reproductive Immunology and Pathology, Institute of Animal Reproduction and Food Research, Polish Academy of Sciences, 10-243 Olsztyn, Poland; m.siemieniuch@pan.olsztyn.pl; 5 The Research Station of the IARF PAS in Popielno, 12-222 Ruciane-Nida, Poland;

**Keywords:** feral horses, welfare, diet, reproduction, management, hoof, insects, parasites

## Abstract

**Simple Summary:**

Feral horses are free to choose their diet, social and reproductive partners, location, and the distance they travel. This behavior and life conditions are often presented as the model for stabled horses’ welfare. However, free-roaming horses are often exposed to conditions or states that may be regarded as welfare threats or abuse. Without human protection, the animals may suffer hunger, thirst, health problems, and aggression from other horses and predators. The aim of this review was to present cases of welfare compromise as well as natural ways to restore high standards of welfare to Konik polski horses (Koniks) living in semiferal conditions in a forest sanctuary over the course of 70 years.

**Abstract:**

To prevent abuse and to assure the welfare of domestic horses, attempts to assess welfare in a standardized way have been made. Welfare-assessment tools often refer to the physical and social environments of feral domestic horses as examples of welfare-friendly conditions for horses. However, free-roaming horses are often exposed to conditions or states that may be regarded as welfare threats or abuse. The aim of this review was to present cases of welfare compromises as well as natural ways to restore high standards of welfare to Konik polski horses (Koniks) living in semiferal conditions in a forest sanctuary over the course of 70 years. Welfare problems in Koniks related to feeding, locomotor, social, reproductive, and comfort behavior, as well as health issues concerning hoof trimming and parasitism in Koniks, are discussed. Periodic food scarcity or abundance, stressful events around weaning and gathering, the consequences of fights among stallions, exposure to sire aggression during dispersal, lameness during “self-trimming,” exposure to insect harassment, high levels of parasitism, and specific landscape formations may endanger free-roaming horses. It has to be underlined that despite the excellent adaptability of horses to free-roaming conditions, one should be aware that welfare problems are to be expected in any semiferal population. Here, we present the management system applied for 70 years in free-roaming Konik polski horses that minimizes welfare threats. It allows close follow-up of individual horses, the strict monitoring of health and welfare on a daily basis, and if necessary, instant reactions from caretakers in cases of emergency. Moreover, it addresses the problem of starvation due to overgrazing and thus, the ethical controversy related to the eradication of surplus animals causing environmental damage.

## 1. Introduction

Nowadays, the welfare of horses used by humans is the subject of concern from horse enthusiasts, animal protectionists, and general society [1,2]. Mainly, controversy related to living conditions and inhumane treatment provokes public emotions. For working horses, i.e., leisure and sport mounts as well as draft equines, aside from the quality of their living conditions, the techniques used as well as the intensity of work are of primary importance for their welfare. Horse welfare compromise has been addressed in studies considering inappropriate feeding [3,4] and keeping conditions [4], restriction of movement and social needs [4,5], and practices that constitute physical and psychological [6,7] abuse. 

To prevent the abuse and to assure the welfare of domestic horses, attempts to assess welfare in a standardized way have been made [8,9]. Several welfare-assessment protocols and welfare-related guidelines were developed for horses [8,9,10,11,12]. Some tools were based on the concept of Five Freedoms of Welfare (from hunger and thirst, from discomfort around resting, from pain and injuries, from fear and stress, and to express natural behavior [13]), adopted as the base for principles and criteria of welfare in the Welfare Quality^®^ framework [8,9]. More recent approaches like The Five Domains model includes four interacting physical/functional domains of welfare (nutrition, environment, health, and behavior) and a fifth domain of mental state (affective/mental experience) [10,14,15].

Welfare assessment tools often refer to the physical and social environments of feral domestic horses (*Equus caballus*) as being natural, welfare-friendly conditions for all horses [16]. However, free-roaming horses are often exposed to the conditions or states that may be regarded as welfare threats or abuse. Without human protection, the animals may suffer hunger, thirst, health problems, and aggression from other horses, predators, or humans. The opportunity to observe and record the feeding, locomotor, social, reproductive, and comfort behavior of Konik horses, as well as the necessity of addressing current health and welfare problems in free-roaming groups, provided us with knowledge regarding potential compromises in the welfare of the monitored horses.

The aim of this review was to present cases of welfare compromise as well as natural ways to restore the high standards of welfare to Konik polski horses (Koniks) living in semiferal conditions in a forest sanctuary over 70 years. In this paper, we utilized the records of 108 horses (84 mares and 24 stallions) that lived and still live in the forest sanctuary in Popielno Research Station, Poland. The observed welfare components related to feeding, locomotion, social, reproductive, and comfort behavior, and health are discussed, and the proposition of an efficient, environment- and welfare-friendly management system for free-roaming Koniks is presented. For the purpose of this review, the literature database PubMed, ISI Web of Science, Scopus, and Google Scholar were searched for “feral horses” and “welfare” (resulting in 40 references), “Konik” (44 references), “reproduction” (56 references), “management” (32 references),“pest” (112 references), “coat” (5 references), “hoof/hooves” (11 references), and “parasite” (20 references). The abstracts of the articles were then inspected for direct relevance to described cases concerning the welfare of free-roaming Koniks and reduced if the search fields overlapped (as for instance “reproduction,” “pest,” and “welfare”). Furthermore, the Konik horses’ bibliography, as well as the records kept by two of the coauthors (Z.J. and M.S.) over the last 33 years of managing the Koniks’ population, was used in the present work. Finally, we used 95 references.

## 2. Konik Horses: History in a Nutshell

Wild forest tarpan horses (*Equus caballus gmelini Ant.*) were last seen in the natural environment in Poland in the 18th century [17]. From this time, tarpans have technically been extinct, but hypothetically, they can be seen to have been crossbred with domestic horses. The project of recreating tarpan-like horses started in the 1920s in Poland and resulted in the creation of the Konik polski breed of horses [17,18]. The most phenotypically tarpan-like horses, with primitive characteristics including a mouse coat with no white markings, zebra stripes on the legs, and a dorsal stripe, were selected from the population suspected of being crossbred with tarpans and constitute the parental material for all Koniks. Since the Second World War, the Konik horses have been kept in two management conditions: in traditional stabling (“stabled Koniks”) and in naturalistic conditions in semiferal/free-roaming groups (“forest Koniks”) [17].

Thanks to the robustness and high adaptability to different habitats, Koniks have been introduced to various environments to maintain the biodiversity and prevent the encroachment of undesired vegetation [19,20,21,22], as elements of “naturalization” of European plains [21,22,23], or to crossbreed with other local primitive breeds like Dülmener horses [17]. The Koniks have also been exported to be used in several environmental projects in Europe (Table 1).

Popielno Research Station is situated on the Popielno Peninsula (53.754556, 21.628361) and is surrounded by lakes on the West, North-East, and East sides. The area available to free-roaming horses is about 1620 ha. The land has a glacial formation with frontal and ground moraines. The area is covered with rush (*Phragmitetea, Scheuchzerio-Caricetea fuscae,* and *Oxycocco-Sphagnetea* classes) and grass communities, xerothermal grasses and ruderal communities (*Molinio-Arrhenatheretea*, *Festuco-Brometea* and *Rudero-Sacalietea* classes), osier (*Salicetum pentandro-cinereae*) and forest communities (*Ribo nigri-Alnetum*, *Circaeo-Alnetum*, *Tilio-Carpinetum*, *Serratulo-Pinetum*, *Querco-Piceetum*, *Vaccinio uliginosi–Pinetum*), with *Betula pubescens* dominated by *Molinia caerulea* and *Peucedano-Pinetum* habitats [28].

The first four mares and a single stallion were released to the area in 1955 [17], and from this time on, the free-roaming population has increased but remains at a stable level of about 20 individuals. The horses were free to choose social and reproductive partners, food, location, and they followed daily, monthly, and yearly cycles of changing natural conditions.

## 3. Monitoring of Free-Roaming Konik Horses in Popielno Sanctuary

The Konik horses in Popielno sanctuary were inspected daily or every other day by the breeding manager (ZJ, MS). Every individual’s birth and death date, cause of death (if known), noticeable health problems (e.g., lameness and injuries), and abnormal behavior (e.g., apathy, nonresponsiveness, and rejection of neonates) were recorded. Human intervention was, however, reduced to the minimum. The caretakers intervened only when the life of the horses was endangered and the necessary veterinary inspection and assistance was provided. Since the stabled group of Koniks was maintained in traditional stables outside the sanctuary, the facilities and staff provided the necessary help in cases of emergency. Thus, the visual control of horses on a regular basis enabled the monitoring of reproduction performance and identification of health problems. Despite such strict surveillance, various accidents and injuries caused health problems or deaths in free-roaming Koniks. The simple descriptive statistics of the monitored population’s life expectancy, reproductive performance, and causes of death (if known) for adults and foals are presented in Table 2.

## 4. Welfare of Koniks Related to Feeding, Drinking, and Locomotor Behavior

Searching for food and its consumption take 70% of the equine time budget and is the main activity of adult horses [29]. Free-roaming horses enjoy choosing their diet according to individual preferences and food availability. Although the horses are preferential grass eaters, in seasons with high food abundance, they choose diverse plants, legumes, green and fallen leaves, tree bark, and even mushrooms. The botanical composition of the diet of Konik horses in Popielno sanctuary assessed by diaspore content in the feces included *Poa* sp., *Rumex obtusifolius*, *Plantago major* ssp. *major*, *Sagina procumbens*, *Festuca rubra*, *Juncus* sp., *Deschampsia* sp., *Carex* sp., *Agrostis* sp., *Plantago lanceolata*, *Rununculus* sp., *Cerastium vulgatum*, *Stellaria* sp., *Trifolium* sp., *Holcus* sp., *Polygonum hydropiper*, *Veronica arvensis, Geranium pusillum*, and *Spergula arvensis* [28,30]. It has been confirmed that in semiferal horses, the intestinal microbial biodiversity is high, which is a positive effect resulting from the diversity of plant species in the diet [31,32].

In winter, especially when the snow covers the ground, Konik horses search actively for food, digging in the snow. They eat dry grass, fallen leaves, raspberry twigs, and birch and willow bark [33]. When the temperatures drop below 0 °C and ice covers the lakes, the horses eagerly enter frozen lakeshores searching for reeds [33]. Searching for food drives the horses to explore dangerous areas. They enter the marshlands where, especially in spring, the reeds have green shoots. Between 1959 and 2020, 17 Koniks drowned in the marshes, lakes, and canals. Although most horses do not enter the marshes, unfortunately, some horses do not learn to avoid them. This is exemplified in the case of one mare, who was found in the marsh and rescued, but drowned in the same place 2 weeks later [33]. This is an example of natural environmental threats that in specific cases may seriously affect the welfare of horses.

In winters with heavy snowfall, the Koniks were occasionally provided with hay of medium nutritional value which they shared with other herbivores present in the forest. However, as the snow melted, the Koniks chose not to feed on hay, preferring dead plants in the forest and forest meadows [33]. Similar to what is concluded in other studies [34,35,36,37,38], the body condition was the poorest in early spring, especially in lactating mares and yearlings. However, it was shown that although forest-reared yearlings were characterized by a lower weight compared to their stable-reared counterparts, their ability to compensate the weight and height resulted in no differences between the 2 years olds from the stable and the forest [39]. The ability to store body fat, characteristic to all primitive breeds [34,35,36,37,38,40], allows the Koniks to survive the seasonal deprivation of quality food successfully. 

Despite the unrestrained access to grazing, which results in quick improvements in body condition in late spring, only two cases of laminitis were observed in forest Konik horses over the last 30 years. Interestingly, these cases, which were subjected to a veterinary consultation, concerned nonlactating mares, who presented with a typical laminitis posture. This problem recurred in one mare in seasons when the mare was barren [33], thus confirming the higher susceptibility of mares to laminitis than stallions [38]. In Australian studies, despite no visible symptoms of laminitis, morphological and histopathological postmortem examinations of hooves evidenced laminar rings in 80% [41] and chronic laminitis in 25% of the feral horses studied [42]. The starvation periods in winter lead to decreased body condition, and recurrent summer obesity may be controversial from the welfare standpoint, with both states being negatively scored in welfare protocols [8,9]. However, it may be hypothesized that, similar to wild herbivores [43], the metabolic mechanisms in free-roaming primitive horses may follow an annual cycle of gaining and losing weight, without negative effects for health and reproduction. Thus, when assessing the welfare of semiferal horse, this ability is proposed as being a natural counteraction to adiposity and acute laminitis [44]. 

Free-roaming horses drink from natural sources of water, such as lakes, ponds, rivers, and human-made reservoirs and canals. They can travel distances up to 70 km to satiate thirst [45]. In winter, the Konik horses lick snow or break the ice with their hooves [33,46]. It was observed that in winter, the Koniks were drinking more frequently (3.1 ± 1.7 times/24 h) than in summer (0.6 ± 0.6 times/24 h), probably resulting from the higher content of dry matter in the forage, but the drinking bouts were shorter [46]. Furthermore, it was observed that on very hot days, especially during periods of high insect harassment, the Koniks entered the deep woods and traveled to waterholes only once a day [47].

Freedom from hunger in semiferal horses may be drastically compromised when the available area that provides the resources for horses and other herbivores is not able to restore because of overgrazing. Uncontrolled increase in the population living in a limited area is the main cause of overgrazing and may result in the death of feral horses due to starvation [21]. In addition, damages to the natural environment caused by feral horses (trampling, tree-bark striping, and decrease in biodiversity [48]) led to the adoption of laws considering horses as pests in Australia and the United States [49,50], and as pests in Australia, they can be exterminated [51]. Usually, the horses are chased by helicopters and cars [52], and then the distinctive harems are corralled (gathered) in one group of stallions, mares, and foals [53]. The procedure is aversive, and alternative methods of gathering, such as a light aircraft leading the horses into a trap, are promising ways to lower gathering stress [53]. The fate of the animals after trapping varies. Although some of the animals are adopted, some of them are culled [50,54,55]. For this reason, in restricted areas like in Popielno sanctuary, the management of the population involves the annual removal of weanlings. This procedure assures a mean stocking rate of 0.012 individuals per ha and allows the habitat to restore. Over 70 years of Konik breeding, no damage to the natural environment due to overgrazing was observed [33].

In forest Konik husbandry, the trapping and weaning/separation of foals is also a very stressful procedure, as confirmed by high fecal cortisol metabolite levels and stress-related behavior, including pseudodepressive states in weanlings [56]. Although closing the bands in traps is noninvasive, as the horses voluntarily enter the enclosures tempted by the hay, the separation of the youngstock from the dams (and the bands), which involves human manipulation, restraint, and transportation to the stable, is one of the most difficult stages in young horses’ lives, more difficult than what stable-reared Koniks experience [56]. Furthermore, later in stable life, the forest-reared foals react with higher levels of fear and aversion to human manipulation during routine husbandry procedures than stable-reared Koniks [18,57]. Thus, high standards of welfare of free-roaming horses may be questionable in these cases. 

When grazing, the horses slowly move by step-by-step locomotion. This active movement takes the horses most of their daily time budget [29,45,46,58]. The motivation to change locations for grazing and drinking can cause the horses to travel up to 70 km [45]. In Popielno sanctuary, the average distance traveled daily (calculated from a monthly measured total) was 12.9 km [28,58]. The distance traveled by a chosen band observed in an annual cycle was not directly correlated with the climatic conditions, such as temperature and precipitation; however, the principal component analysis showed that there was increased locomotor activity as the precipitation increased [58]. Nevertheless, the season was not found to be significant, which shows that the need for locomotion in Konik horses is independent from time of the year [58]. 

## 5. Welfare of Koniks Related to Social and Reproductive Behavior

**Figure 1:**
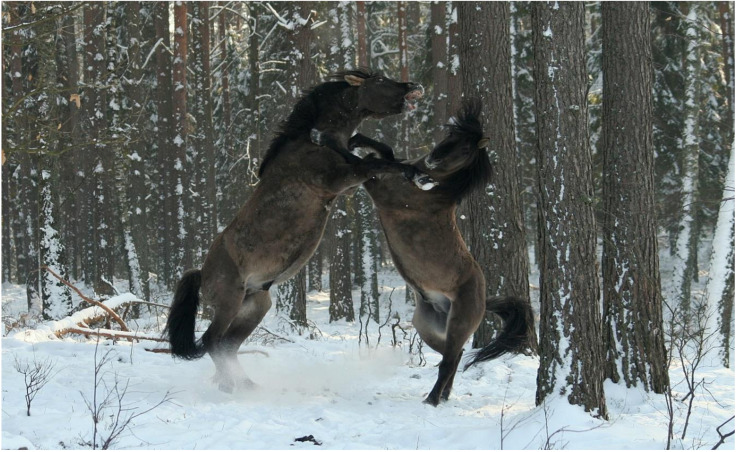
Fighting Konik stallions (photo by Michał Bruzda).

The social behavior of the Koniks did not differ from other free-roaming horses [33]. However, no tendency for grouping into big herds [23] was observed, with one exceptional case of two stallions (out of 21 that were able to keep their harems; data for 1959–2020) that usually roamed together with their harems. The sole but important difference was the annual reduction in the population by removing the weanlings. Hence, the composition of bands was skewed for higher female representation, and no bachelor groups were ever formed in Popielno sanctuary, as observed in other feral herds [23,59]. This policy had an impact on the social behavior of horses, as from the very beginning, the stallions could easily form and maintain harems, and unlike in multistallion populations [23], no harassment from young stallions ever took place [60,61]. Despite this comfortable situation, fights between harem stallions occurred during and outside the reproductive season [59]. The take-over of harems due to fights usually resulted in the quick death of the defeated stallion as a result of the exhaustion caused by the constant alertness, decreased feeding time, and harassment, and most probably, the on-going health problems which could have been the primary cause of the gradual loss of stallion’s power to guard the mares and succeed in fights [59]. Although confrontations between stallions in their prime mostly consisted of impressive parades [60] and resulted in rather superficial injuries, they were sometimes very serious (Figure 1). A case of a potentially fatal fight resulting in a mouth injury (a piece of lip was bitten off, preventing the animal from feeding) was recorded. This is another example of how the welfare of free-roaming horses may be impaired.

The social hierarchy in Konik bands was similar to other free-roaming horses [62]. The strong bonds between nonrelated adult females, usually lasting their whole life [59], are typical of horses [63]. It was observed that after the harem stallion’s death, Konik mares stayed together in the absence of the next harem stallion for 6 months [59]. Every new mare was initially chased off by resident mares, but finally, especially after foaling, entered the group [33]. Mating in Koniks was restricted to the harem, and contrary to multistallion feral populations [64], no sneak copulations occurred in Popielno sanctuary, as confirmed by the parentage testing [59].

As in other feral populations, the foals left in the sanctuary for parent replacement were chased off by the harem stallion [64], which is assumed to be a behavioral mechanism to prevent inbreeding [65]. Although this paternal behavior is natural, it may be aggressive and skin injuries were frequent in chased offspring. In view of the scarcity of counterparts, expelled young Konik horses usually roamed solitarily before they were incorporated into another harem (fillies) or until they were able to fight for their own mares (colts). Solitary stays in the forest are not always safe. Data from the period 1986–2000 show that 6 out of 24 (25%) young expelled horses did not survived this period for various reasons, including unfortunate accidents [33]. Although no depredation for adult Konik horses in Poland was confirmed (except for one suspected case, Table 2), when packs of wolves become more numerous in the region, the life of a solitary horse may become more precarious. 

**Figure 2:**
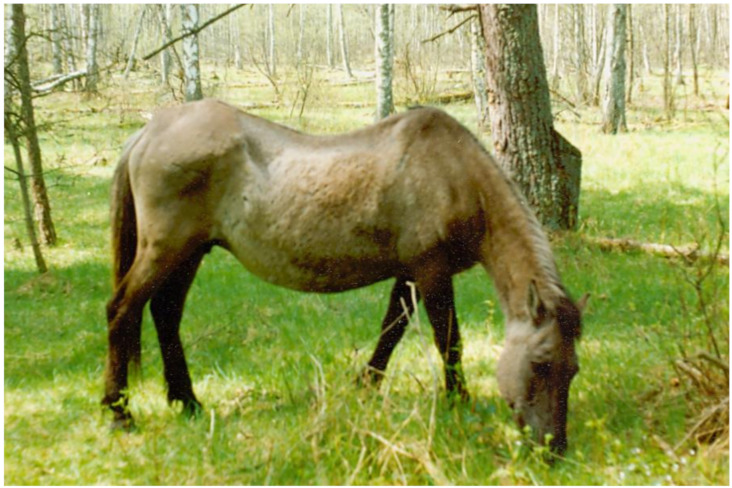
The oldest mare in Popielno sanctuary as a 29-year-old (photo by Zbigniew Jaworski).

Koniks have to a 96.5% foal survival rate up to one year old (Table 2). It is important to note that the reproductive history of certain mares in the sanctuary may be very long, with one exceptional mare (Figure 2) producing 25 foals for 30 years, with only two barren seasons. Geriatric mares were present in the band until their death, with the last seasons being barren [59], which decreases individual lifelong reproductive performance. Furthermore, fatal cases related to dystocia (including uterine torsion), wolf predation during delivery (suspected), and congenital defects in foals were recorded (Table 2). Nonetheless, the excellent reproduction of feral horses, as compared to their stabled counterparts, even in periodic starvation conditions, is a phenomenon that indicates the detrimental effects of genetic selection and the breeding conditions in stabled horse populations [66,67]. 

In Konik sanctuary in Poland, there is no need for the culling of unwanted animals. For other populations of feral horses, immunocontraception is another more humane counterstrategy to high reproduction rates and burgeoning feral populations [55,68,69,70,71]. However, the long-term effects of immunocontraception include the return to fertility after three to seven seasons [70], and in some cases, higher rates of reproductive behavior in treated mares were observed [71]. 

## 6. Welfare of Koniks Related to Comfort Behavior

The performance of comfort behaviors in horses living in naturalistic conditions is not restrained. Konik horses could roll, scratch, enter the water, and mutually groom which promoted good skin and hair conditions. In the North-East European climate, the Koniks seasonally change their coat. The winter coat of horses from the sanctuary was significantly denser than in the stabled group and its density increased with the age of the horse [72].

For rolling, the horses chose sandy places, which were regularly used by all horses in the band in a ritualized way, starting with the highest ranked individuals [35]. The horses could also dig for new “sandpits” [35]. They also willingly rolled in snow, however, when rolling in very thick snow, heavily pregnant mares risk entrapment in the dorsal position ([35] Table 2). Similarly, in areas where deep furrows are found in the forest, cases of horses becoming stuck when rolling were observed [35]. 

Scratching is a behavior Koniks eagerly performed, especially in days of intense insect harassment [47]. The tail-to-head position and bunching in a group with heads in the center of the bunch help free-roaming horses provide mutual protection against the insects [47,73,74]. Since the Koniks have no shelters in the sanctuary, they chose to stay in dense forests during the hottest times of the day [47,73,74]. The horses changed refuges every day, and it was observed that separate bands use such places interchangeably [33]. It has to be noted that without such naturally shadowed refuges, the horses would be much more exposed to hot weather and insects [47].

## 7. Health Aspects

**Figure 3:**
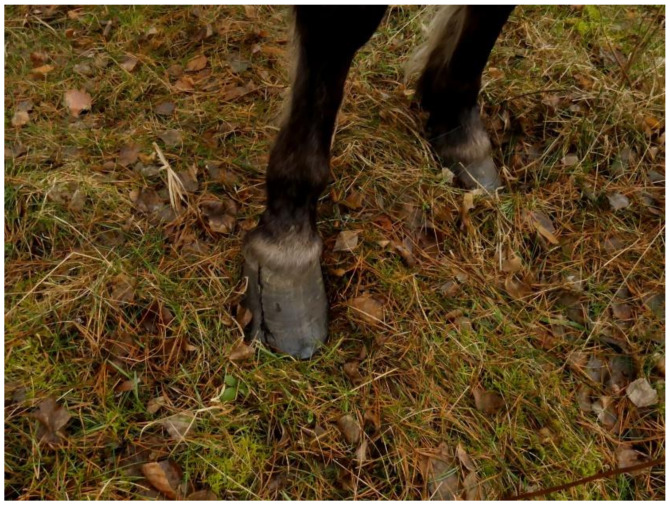
Cracked hoof in a Konik mare (photo by Marta Siemieniuch).

Since Koniks are isolated from other conspecifics, no infectious illness was diagnosed in this population. Most health problems were related to hoof wear and parasite invasions. The Koniks in the sanctuary were not trimmed and their hooves were subjected to the natural processes of growing and shaping during wearing, which relate to season, distance traveled, and ground specifics [34,74,75,76,77,78,79]. Within sanctuary borders, Koniks usually travelled on grassy or sandy ground, and analyses showed that hooves were not worn off as a whole [78]. Actually, the horny part of the hoof is torn and distorted as it becomes overgrown [77,78]. Before the horn detaches from the hoof wall, the wall cracks about 1–3 cm [77,78]. No deformation of the hooves or leg posture was noticed in Koniks. It was observed that after 1–3 months, the hoof looked normal, and with this natural “self-trimming,” no lameness was ever observed during the daily visual inspections, which included an inspection of any internal abnormalities of the hoof [41,76,80]. In the last 30 years, cases of deeper cracks, up to the middle of the hoof or even up to the coronary band (Figure 3) were noted in three horses [77]. Even so, no lameness was observed in two of the horses. In the third horse, lameness lasted 2 months: the horse was not able to stand on the affected leg, but as soon as the fractured horn had broken, the horse became sound within 1 week [77]. Usually, the cracks were not deep and naturally filled in. Interestingly, it was found that the hooves of “forest” Koniks grew at a slower rate than those in a “stable” group, where the horses are regularly trimmed [77] and followed seasonal changes, similar to other feral domestic [75,78] and Przewalski (*Equus ferus Przewalskii*) horses [81]. Generally, periods when hooves were in a better and worse condition occurred spontaneously during the horses’ lifetime [77]. This can be explained not only by periodic (winter) drops in food quality and availability but also with more regular locomotion on varied types of ground [76,80]. Thanks to slow locomotion and no predation, lame horses can survive; nonetheless, their welfare is reduced during such critical periods. However, a lack of lameness is not evidence that the hooves are healthy, since lameness may not occur during the on-going laminitis [38,42] or self-trimming [76,80]. Frequent histopathological changes in feral horses’ hooves observed in post mortem studies indicate that the feral horse foot type cannot be used as a model for the domestic horse foot [41,80].

Another health-related issue in free-roaming horses is parasite infections [82,83,84,85,86,87,88,89,90,91,92,93,94]. In most studies, the body condition of feral horses was not related to the level of infestation [85], although this was not a rule [86,87]. The studies carried out from 1999 to 2007 show that Koniks were infected with strongyles (*Strongylus spp.*, *Cyastoma*), roundworm (*Parascaris equorum*), and tapeworms (*Anoplocephala* spp) [85,86]. Slivinska et al. [91] found that in 2009, strongyles, represented by 24 species (two large strongyles and 22 *cyathostome* species), were present in 100% of forest yearlings, tapeworms (*Anoplocephala perfoliata*) were observed in 72.7% of yearlings, and *Gasterophilus intestinalis* was found in 90.9% of yearlings [91]. The prevalence of *Strongylus vulgaris* was 81.8% [91] and was higher compared to an earlier study (66.7%) [93]. Contrary to the body condition, the level of infection in Koniks was highest in the summer months, and lowest in the winter months [85]. Even though the highest number of strongyle eggs may amount to several thousand per gram of feces [82,88], no case of health problems was observed, maybe thanks to some of the behavioral strategies proposed by Hart [73,74]. One exception was a mare that died 1 month after foaling. The necropsy revealed the perforation of intestines filled with roundworms and larvae of *Hypoderma bovis* [89]. It is supposed that Koniks search for and eat plants with anthelmintic properties, such as floating sweet-grass (*Glyceria fluitans*), bittersweet (*Solanum dulcamara*), caraway (*Carum carvi L.*), greater celandine (*Chelidonium maius*), blueberry fruits (*Vaccinium myrtillus L.*), wild thyme (*Thymus serpyllum*), roots of the male fern (*Dryopteris filix-mas L*.) [89], and the mushroom bitter bolete (*Tylopilus felleus*) [28]. It was suggested that wild horse populations in South-East Australia with a high prevalence of *Strongylus vulgaris* may act as a reservoir of infection for domestic horses [90].

In terms of external parasites, blood-sucking species are the main problem for the horses. On hot days, they entered the lakes, most probably to cool the limbs down, but also, presumably, to alleviate insect bites, such as horse flies (*Tabanidae*), forest flies (*Hippobosca equina*), warble flies (*Hypoderma spp*), face flies (*Musca autumnalis*), stable flies (*Stomoxys calcitrans* L), and the midges (*Culicoides spp*). Harassment from insects affected the diurnal rhythm of horse activity and increased the frequency of defensive behaviors, e.g., tail swishing, head shaking, leg shifting, kicking the belly, and skin twitching [47]. The climatic (wind and temperature) and social (groups size and interindividual distance) conditions affected protective behavior [47,94]. Long manes and tails allowed for the displacement of insects; however, they were also prone to tangling with thistle or burdock (*Carduus spp L.*) burrs, which stayed on the horses until the burrs disintegrate [33].

Ticks (*Ixodes ricinus*) were not very frequent parasites for Koniks; however, cases of piroplasmosis were suspected in individuals found dead without any other obvious cause (indicated in Table 2 as “unknown”). This confirms that semiferal horses are exposed to dangers originating from the changing natural environment, such as the increasing omnipresence of ticks, including those infected with *Babesia equi* or *Theileria equi* [92].

## 8. Management Techniques to Assure Welfare in Semiferal Konik Horses

The welfare of free-roaming horses may be considered very high when compared to some stabled horses, which experience social and maintenance restrictions. It is generally true, however, that welfare can be seriously decreased by food scarcity causing starvation, or abundance causing laminitis, stressful events around weaning or gathering, the consequences of fights among stallions, exposure to sire aggression during dispersal, lameness during “self-trimming,” exposure to insect harassment, high levels of parasitism, and hazardous incidents due to specific landscape formations. 

Surprisingly, free-roaming horses are perceived in extremely different ways: from totally destructive (Australia and the United States) [48,51,95,96] to beneficial (Europe) [19,20,24,25,26,27] for the environment and biodiversity. The management of feral horses provokes debate and ethical concerns [20,21,22,48,51,95,96]. Free-roaming horses experience welfare threats, which are evidenced by examples from bands that are strictly monitored [10]. In the studied population of Koniks, health problems were inspected by a veterinary surgeon. Notwithstanding, it was not possible to prevent all cases of fatality (Table 2).

In Koniks, monitoring was possible only because the number of horses was being kept low. In Poland, the breeding and management policy of the Konik polski breed has not changed for almost a 100 years, and still, it functions very well. The breeding and management of semiferal studs are supervised by the Konik Polski Breeders Association [http://zhkp.pl/], which sets the breeding program, comprising strict rules for the free-roaming horses’ keeping conditions. All horses were identified and entered in the Konik polski studbook. The breeding program focused primarily on the maintenance of the primitive, robust type with the ability to cope with the conditions typical to the primeval forests of East-Central Europe all year round [http://zhkp.pl/informacje-dla-hodowcow]. The breed is involved in a genetic resources protection program through the Convention on Biological Diversity (https://www.cbd.int/).

Husbandry politics for semiferal Koniks populations in Poland presumes the control of the number of reproducing horses. This is made possible by the annual removal of all foals around the natural weaning time, which is the most substantial interference in Koniks’ lives [56]. The procedure of removing the weanlings from the sanctuary was described in detail in Górecka-Bruzda et al. [56]. The weanlings are transferred to stables outside the sanctuary and habituated to humans [57]. Then, they are successively sold as leisure horses or breeding stock.

The procedure is repeated each year unless the breeding manager decides to leave a weanling for the replacement of its parent. This occurs only once, or exceptionally, twice in a parent’s life, and keeps the population at a stable level (around 20 horses). To counteract the increase in inbreeding, horses from other sanctuaries are introduced sporadically. Only animals born wild can be released into the sanctuary. This procedure is very effective in small population management. It has to be admitted that the population of Koniks, although enjoying the full freedom of “wild” horses, was sex-skewed towards females. Without young males, no bachelor stallion bands or multistallion harems were formed in Popielno sanctuary. There were three to five concurrent harems throughout the 70 years concerned. We are strongly convinced that for the sake of the welfare of the horses and for protection of the environment and the wildlife sharing the same area, this procedure is efficient, sustainable, welfare-friendly, and ethically noncontroversial.

## 9. Conclusions

Human interventions preventing decreases in the welfare of feral and free-roaming horses have to be continued for the sake of the animals and for environmental protection. The system, which has been applied for 70 years for the breeding and management of free-roaming Konik polski horses and is approved and implemented on a national level, allows for close follow-ups of individual horses, the strict monitoring of health and welfare on a daily basis, and if necessary, an instant reaction from the caretakers in cases of emergency. Moreover, it prevents the ethical controversy related to the eradication of surplus animals, which are the source of overgrazing and environmental damage. Despite the numerous cases of welfare issues that were observed to resolve themselves naturally, some had fatal consequences. It has to be underlined that despite the excellent adaptability of horses to free-roaming conditions, one must be aware that welfare problems are to be expected in any semiferal population.

## Figures and Tables

**Table 1:** Konik polski horses in European environmental projects.

Country	Designation	Location	References
The Netherlands	Restoration of natural environments with large herbivores	Oostvaardersplassen Blauwe Kamer Nature Reserve	[21,22,23] [24]
Germany	Crossbreeding with Dülmener horses. Restoration of natural environments with large herbivores	Lower Unstrut Valley	[17] [25]
Poland	Genetic resource protection Restoration of natural environments with large herbivores	Popielno Research Station Roztocze National Park Rakutowskie peatbogs Narew and Narewka Upper Valleys Oświn Lake Reserve Biebrza National Park Kliniska Forestry	[17] [17] [19] [19] [19] [19]
Latvia	Restoration of natural environments with large herbivores	Nature Park “Pape”	[26]
Belgium	Restoration of natural environments with large herbivores	Westhoek	[20]
UK	Restoration of natural environments with large herbivores	Wicken Fen	[27]

**Table 2:** Life expectancy, reproductive efficiency of mares, and causes of death of Konik horse stallions, mares, and foals reared up to 1 year old.

	N	Mean	Sd	Me	[Q1; Q3]	Range
Life expectancy—stallions	10 ^A^	19.6	6.6	22	[14; 25]	5–15
Life expectancy—mares	46 ^B^	17.8	9.4	20	[10; 24]	2–33
Foals born ^C^	789	9.4	7.3	8.5	[3; 15.5]	0–25
Foals reared ^C^ (%)	731	8.7 (96.5)	7.0 (18.1)	8	[2; 15]	0–25
Foals lost ^C^ (%)	58	0.7 (3.5)	1.3 (15.5)	0	[0; 1]	0–7
						
Cause of death–stallions	10	Advanced age (N = 7)		Other reasons * (N = 3)		
Cause of death–mares	46	Advanced age (N = 16)	Drowning (N = 10)	Other reasons ** (N = 20)		
Cause of death–foals	58		Drowning (N = 7)	Other reasons *** (N = 51)		

^A^ Per 24 stallions (the total also includes 3 alive and 11 that stayed temporarily); ^B^ per 84 mares (the total also includes 20 alive and 18 that stayed temporarily); ^C^ per mare in observed lifetime. * Other causes of death of stallions include stomach rupture (1 stallion), broken shoulder (probably from a kick, euthanized, 1 stallion), and one stallion disappeared (the body was not found). ** Other causes of death of mares include dystocia (3 mares), uterine torsion (2 mares), inability to stand up (2 mares), internal bleeding due to kick (1 mare), broken leg (1 mare), intestine rupture due to parasite infestation (1 mare), probable wolf predation (1 mare), asphyxiation in a poach (1 mare), unknown reasons/the body was not found (8 mares). *** Other causes of death of foals include congenital defects (9 foals), car accidents (3 foals), stomach rupture (1 foal), unknown/the body was not found (38 foals).
